# Copper metabolism and its implications for canine nutrition

**DOI:** 10.1093/tas/txad147

**Published:** 2024-01-03

**Authors:** Laura A Amundson, Brent N Kirn, Erik J Swensson, Allison A Millican, George C Fahey

**Affiliations:** Zinpro Corporation, Eden Prairie, MN 55344, USA; Animal and Dairy Sciences Department, University of Wisconsin-Madison, Madison, WI 53706, USA; Zinpro Corporation, Eden Prairie, MN 55344, USA; Zinpro Corporation, Eden Prairie, MN 55344, USA; Zinpro Corporation, Eden Prairie, MN 55344, USA; Department of Animal Sciences, University of Illinois at Urbana-Champaign, Urbana, IL 61801, USA

**Keywords:** canine nutrition, copper metabolism, copper-associated hepatitis

## Abstract

Canine copper nutrition has received increased attention due to recent reports of apparent copper-associated hepatitis in the USA and European Union. In order to properly address the need to modify the U.S. National Research Council and Association of American Feed Control Officials canine copper recommendations that will have implications for all dogs, it is important to understand the complexities of copper metabolism, confounding variables affecting copper status, and the available research on this topic in dogs. Recent trends in consumer preference for dog diets, supplements, and functional treats introduce another layer of complexity, as most ingredients used in these formulations provide vastly different proportions of essential nutrients, thus resulting in great variation in nutrient profiles available to the animal. Although controlled research addressing copper metabolism in dogs is limited, there are lessons to be learned from other monogastric species as well as canine case studies that can provide a base for increasing knowledge to address this issue. Copper metabolism and status in animals is affected by a multitude of factors including absorption, storage, excretion, and nutrient interactions. Given its vital role in many physiological processes, it is important that both nutritional deficiencies and toxicities be avoided. Additionally, another challenge for proper copper nutrition in dogs is the known genetic predispositions of some breeds for copper storage and excretion abnormalities. Therefore, it is imperative that veterinarians, nutritionists, and pet food manufacturers collaborate with the shared goal of providing dog food options that supply the essential nutrients at adequate concentrations to support an active and healthy life. Many questions remain regarding copper metabolism and proper diet formulation for dogs. Future research efforts should focus on discovering reliable, non-invasive methods for evaluating canine copper status, a deeper understanding of genetic predispositions of certain breeds, increased knowledge of copper contributions from various ingredients, and the role of unpredictable physiological stressors on copper metabolism.

## INTRODUCTION

Copper nutriture of the dog has recently received increased attention in the USA and European Union due to reports of apparent copper-associated hepatitis (CAH). Recent trends in canine nutrition have led to new questions regarding proper dietary copper concentrations in canine diets. Recently, the Association of American Feed Control Officials (AAFCO) and the U.S. National Research Council (NRC) guidelines for canine dietary copper concentrations have been questioned due to the lack of upper tolerable limit values (Center et al., 2021). Given the reported increase in CAH and the current trends in canine nutrition, these concerns should be investigated. To identify the best course of action regarding these questions, it is important to consider the complexities of copper metabolism, available trace mineral research in dogs and other animal species, dietary ingredient composition and nutrient variability, and the potential effects of different supplemental sources of copper. The objective of this review is to provide basic information about copper metabolism as it is understood in humans and other animal species and provide an overview of the knowledge and questions that remain regarding canine copper nutrition.

Historically, dog diets have been comprised of a mixture of grains, animal proteins, and byproducts. However, recent trends in consumer preference have shifted formulations towards grain-free diets containing higher concentrations of protein that has led to increased inclusion of novel ingredients including pulses, fresh meat, and organ meats, such as liver, in complete and balanced dog diets and treats. Additionally, raw meat and homemade formulations are gaining in popularity. Unsurprisingly, these dietary ingredients inherently provide different proportions of essential nutrients and, thus, result in different nutrient profiles for the animal. One of the essential nutrients that has recently come under greater scrutiny is copper due to its higher concentrations in these novel ingredients, as well as the inclusion of more bioavailable forms of copper in supplements and functional treats.

While there is limited scientific research in dogs to evaluate requirements and establish upper tolerable levels of most nutrients, it is imperative to review what research has been done in canine case studies and controlled studies in other species to identify what is applicable to dogs as well as in what areas questions and concerns remain. The following is a brief review of copper metabolism in monogastric animals and considerations that need to be understood when formulating dog diets to meet physiological requirements and assess the risk of potential toxicity. Focus should be placed on increasing our knowledge and understanding of copper metabolism by dogs before making changes in recommendations that could have broad implications for all dogs.

## COPPER METABOLISM: ABSORPTION, DISTRIBUTION, STORAGE, AND EXCRETION

Copper is an essential nutrient required by mammals due to its variety of roles in physiological processes necessary for basic function and health. However, given its chemical properties as a transition metal, concentrations need to be adequately balanced in diet formulations. Copper can be an acceptor or donator of electrons due to its two oxidation states, cuprous (Cu^+^) and cupric (Cu^2+^) copper. Therefore, an excess of copper in cells could be detrimental due to potential free radical formation and subsequent oxidative stress (Chen et al., 2020). However, the capability of copper to switch readily between oxidation states is responsible for its essential role in a variety of enzymatic and biochemical reactions. Notably, these include cytochrome *c* oxidase (electron transport chain function), lysyl oxidase (collagen and elastin formation), Cu/Zn superoxide dismutase (SOD; antioxidant defense), dopamine beta hydroxylase (neurotransmitter biosynthesis), tyrosinase (pigmentation), sulfhydryl oxidase (keratinization), and ceruloplasmin and hephaestin (iron homeostasis; Goff, 2017; Møller and Aaseth, 2022). Given copper’s role in these various enzymes, the micronutrient is integral in ATP production, connective tissue growth and development, bone and cartilage formation and function, antioxidant reactions, iron metabolism, and antimicrobial functions (Wu, 2018).

Below is a brief synopsis of the general aspects of copper absorption, storage and distribution, and excretion as well as common copper deficiency and toxicity symptoms to support the objective of this review. A more in-depth publication on copper metabolism and physiology that is beyond the scope of this review is presented in *Principles of Animal Nutrition* (Wu, 2018).

### Absorption

Circulating copper (Cu^2+^) is sensed by intestinal enterocytes and converted by brush border reductase enzymes to Cu^+^, the form in which it can be absorbed by the enterocyte. The copper transporter 1 protein (CTR1) transports Cu^+^ across the apical membrane of the enterocyte. Small amounts of Cu^+^ can be transported into the enterocyte via divalent metal transporter 1. Copper is immediately bound by chaperone proteins, glutathione (GSH), or metallothionein (MT), to reduce the risk of oxidative damage. The GSH-bound copper will subsequently be transferred to copper chaperone proteins that carry Cu^+^ to various cellular compartments or proteins responsible for export based on cellular and systemic copper needs (Kaplan and Maryon, 2016; Goff, 2017; Chen et al., 2020).

Cellular copper chaperones include copper chaperone for SOD that carries Cu^+^ to SOD, a pivotal enzyme involved in antioxidant defenses; cytochrome *c* oxidase 17 that carries Cu^+^ to the mitochondria for proper functioning of cytochrome *c* oxidase, an indispensable component of the electron transport chain and cellular energy metabolism; and antioxidant 1 (ATOX1) that carries Cu^+^ to ATPase copper transporting alpha (ATP7A) in the enterocyte or ATPase copper transporting beta (ATP7B) in the hepatocyte (Goff, 2017; Chen et al., 2020).

Once Cu^+^ is bound to ATP7A, it is transported through the cell via a transport vesicle to the basolateral membrane and subsequently released into circulation, bound to circulating proteins, mainly albumin, and transported to other tissues, mainly the liver. The liver is the main organ responsible for maintaining copper homeostasis. Hepatocyte reductase enzymes reduce circulating Cu^2+^ to Cu^+^ and transport it into the cell via CTR1. Once in the hepatocyte, the same chaperone proteins present in the enterocyte will shuttle copper to the necessary cellular sites for enzyme function, protein synthesis, or excretion depending on cellular and systemic needs (Goff, 2017).

### Storage and Distribution

The liver is responsible for synthesizing copper-containing proteins for transport to other tissues. This is facilitated via the hepatic ATPase, ATB7, present in the Golgi membrane, the protein factory of the cell. The major copper-containing protein produced in the liver is ceruloplasmin. Ceruloplasmin can carry copper to cells throughout the body. Additionally, ceruloplasmin is a ferroxidase enzyme. Ferroxidsae oxidizes ferrous (Fe^2+^) to ferric (Fe^3+^) iron which is necessary for maintaining iron homeostasis. Approximately 40% to 70% of plasma copper is bound to ceruloplasmin (Goff, 2017).

Copper homeostasis is maintained via absorption efficiency, sequestration, storage, and excretion. When the animal has adequate copper stores, enterocyte CTR1 will be internalized and recycled for later use or degraded (Chen et al., 2020). At the same time, enterocyte MT production increases to sequester excess Cu^+^ and prevent enterocyte oxidative damage until it can transfer the Cu^+^ to ATOX to be incorporated into ATP7A for export to the liver for storage or excretion. Copper that remains bound to MT when an enterocyte dies and is shed will be excreted in the feces (Goff, 2017). Normal circulating and liver copper concentrations are presented in Table 1 (Puls, 1994).

**Table 1.** Normal liver and serum copper concentrations in animals (ppm)^1^

Tissue	Animal				
	Cattle	Sheep	Pigs	Chickens (*Growers*)	Dogs
Serum	0.60 to 1.50 (*no supplemental Se)*	0.70 to 2.00	1.30 to 3.00	0.08 to 0.50	0.20 to 0.80
Liver (wet weight)	25 to 100	25 to 100	5 to 100	3 to 15	30 to 100

^1^ Puls (1994).

### Excretion

The liver has a large capacity to store copper, but once that capacity has been met, ATP7B transports excess Cu^+^ out of the liver where it is excreted via bile. An animal’s capacity for biliary excretion of copper is what determines its copper tolerance and is responsible for the vast differences in animal species’ risk for copper toxicity. Even though liver copper storage capacity is high, there are events that increase the risk for local and systemic oxidative damage due to Cu^+^ release. During physiological stressful events (i.e., inflammation and infection), liver protein turnover increases and has the potential to release the stored, highly pro-oxidant Cu^+^ locally and systemically, increasing the risk for widespread oxidative damage, ultimately causing cellular and tissue death.

### Deficiency and Toxicity

Common signs of copper deficiency include loss of hair color, reduced fertility, impaired cellular immune response, and impaired connective tissue integrity. These deficiencies are not surprising given copper’s role in multiple enzymes specific to these physiological functions and its indispensable role in cellular energy homeostasis. Immune function may be impacted by a copper deficiency before more common signs of deficiency are obvious. For example, a study in sheep suggested that Cu requirements increase during an immune challenge (Suttle, 2012). Additionally, ceruloplasmin, MT, and other copper-containing acute phase proteins increase during inflammation and infection that will result in increased binding of Cu and subsequent risk for secondary deficiencies due to the copper being unavailable to the animal for cellular and tissue homeostasis.

Common consequences of copper toxicity include decreased liver function, hemolysis, and cellular and tissue necrosis. Stress, inflammation, infection, or other immune challenges can result in increased liver protein turnover and increase Cu^+^ release into the circulation where it can overwhelm carrier protein capacities and result in widespread cellular and tissue damage due to its strong pro-oxidant properties (Goff, 2017).

Both deficiency and toxicity signs can be delayed relative to the onset of innate or induced copper imbalances based on the nature of the perturbation. For example, deficiency signs may be delayed due to a lag between reduced copper concentrations and copper stores being depleted. At the same time, toxicities can be acute or chronic. Chronic copper toxicity can occur due to exposure to elevated dietary copper for long periods of time without signs of toxicity until the storage capacity of the liver is overwhelmed. Acute copper toxicity can occur after a physiologically stressful event (i.e., inflammation and infection) where liver protein turnover is elevated and a large amount of Cu^+^ is released.

## CONSIDERATIONS FOR BALANCED COPPER NUTRITION IN DOGS

Reports of CAH and inflammatory hepatic disease in dogs have increased over the last two decades. While the risk of toxicity from copper is elevated compared to other micronutrients, it is vital to understand the various factors that affect copper metabolism to formulate canine diets that meet the animal’s requirements while minimizing the risk for toxicity, given the important physiologic roles of copper mentioned earlier.

There are two important factors to consider regarding canine CAH when determining recommendations for dietary copper inclusion: genetic predisposition and environmental influence (i.e., diet).

### Genetic Predisposition to Copper Toxicities

Bedlington Terriers were the first breed to be recognized as having a causational mutation leading to CAH. A mutation in *COMMD1* led to impaired biliary excretion of copper, thus causing the liver to be overwhelmed by Cu^+^, leading to hepatic pathologies (Dirksen and Fieten, 2017). Labrador Retrievers were recognized as having a genetic component of CAH risk, but it appeared to be much more complex than was the case for Bedlington Terriers. Only 12% of the heritability of CAH in Labrador Retrievers can be accounted for by genetic mutations identified to date and, therefore, environmental and/or other yet to be identified genetic factors are at play (Johnston et al., 2013; Dirksen and Fieten, 2017; Strickland et al., 2018; Wu et al., 2020). There are breeds other than Bedlington Terriers and Labrador Retrievers that have been identified as having suspected hereditary CAH and are, therefore, considered to be predisposed to copper toxicity and CAH. These include West Highland Terriers, Doberman Pinschers, and Dalmatians (Spee et al., 2005; Johnston et al., 2013; Dirksen and Fieten, 2017; Strickland et al., 2018).

In a recent retrospective study of 546 dogs, Strickland et al. (2018) reported evidence for significantly increased hepatic copper concentrations detected in dogs over the years of 1982 to 2015. This increase was not limited to dogs of breeds considered to be predisposed to CAH. The cutoff values for hepatic copper concentration used in this study were 300, 400, and 1,000 μg Cu/g liver (dry weight basis) based on when the hepatic injury was likely and individual clinic reference ranges. Importantly, the authors point out that normal hepatic copper concentrations for dogs are not clearly established and that the definitive clinical relevance of the observed increases in hepatic copper concentrations remains elusive (Strickland et al., 2018). Unfortunately, the dietary histories of these dogs were not available in this analysis.

Breeds that are predisposed to hereditary CAH may serve as good models to understand how diet may be used as a therapeutic agent to prevent and/or treat risk of serious liver pathologies. There is some evidence that feeding lower copper diets (~4.8 ppm Cu on a DM basis) to Labrador Retrievers may decrease liver Cu concentrations by roughly 36% (Hoffmann et al., 2009; Fieten et al., 2012). This area warrants further investigation.

### Dietary Contributions to Copper Toxicity

It has been speculated that the increased incidence of CAH cases coincided with AAFCO recommendations to stop the use of copper oxide as the source of supplemental Cu in dog diets due to its extremely low bioavailability (Strickland et al., 2018; Center et al., 2021). This speculation raises questions about the bioavailability of copper in various dog dietary ingredients and copper supplementation sources, as well as how those factors are affected by other nutrients and different physiological states. The NRC (2006) recommendation for adult dogs at maintenance is 6 ppm Cu (total diet) based on a dietary energy concentration of 4,000 kcal (DM basis) with higher concentrations recommended for puppies after weaning (11 ppm, total diet) and females during late gestation and lactation (12.4 ppm, total diet). Minimum inclusion levels recommended by AAFCO (2023) are 7.3 ppm (total diet) for adult dogs at maintenance and 12.4 pm (total diet) for growth and reproduction periods or for all life stages (DM basis). However, no upper tolerable limits have been established by either organization. It is important to distinguish that these recommendations are for minimum total copper concentrations, i.e., dietary ingredient plus supplemental (premix) contributions, not minimum supplemental concentrations.

Copper bioavailability in ingredients used in dog diet formulations has, for the most part, not been studied in dogs and is generally unknown. However, extrapolations from other monogastric species have been made, mostly from chickens and pigs. These diets are mainly composed of corn and soybean meal and even though ingredient inclusion is somewhat consistent across the industry, there is still a large amount of variation and inconsistency in estimates of Cu bioavailability. In general, copper derived from common feed ingredients and additives in chicken and pig diets is considered to have a relative bioavailability of 50% compared to a copper sulfate standard, with a wide range of 10% to 50% being suggested in swine diets (EFSA, 2016).

As mentioned earlier, there is an increased prevalence of raw meat and grain-free/high protein-based diets for dogs. Organ meats are inherently high in copper concentration and, therefore, the relative bioavailability of that copper needs to be considered and accounted for. Copper concentrations are higher in the liver of ruminant species like beef and lamb but are much lower in chicken and turkey liver. There is also potential for variable bioavailability based on the form of liver used (i.e., freeze dried or fresh) as well as the plane of nutrition of the animal that the liver was derived from. Aoyagi et al. (1993) utilized a chick bioassay to determine the relative bioavailability of copper from the freeze-dried liver of different animal origins. The following bioavailability values (%) relative to copper sulfate (100) were determined: 0 (pork), 21 (rat), 82 (beef), 83 (turkey), 113 (sheep), 116 (chicken—low Cu), and 135 (chicken—high Cu). Additionally, they concluded that when fibrous ingredients (peanut hulls or soy mill run) were present, the bioavailability of copper sulfate was decreased by roughly 50% (Aoyagi et al., 1993). This study provides pivotal baseline information that can be used when formulating dog diets with increased inclusion of liver.

Taken together, copper bioavailability in common feed ingredients in monogastric livestock species is variable and inconsistent. As exemplified in Aoyagi’s study, animal-derived high copper-containing feed ingredients, like liver, vary by species origin. While dogs are a monogastric species with a similar digestive system to pigs, canine-specific factors need to be considered when extrapolating bioavailability data. These include but are not limited to diet complexities, growth rates, and passage rate of different diet matrices. There is limited research in dogs related to ingredient copper bioavailability and, therefore, further research is warranted to try to better estimate relative bioavailability that can be directly applied in canine nutrition.

Another important factor to consider when formulating dog diets is the interaction between nutrients and potential antagonisms that can occur. It is imperative that both dietary ingredients as well as supplemental contributions are taken into consideration when formulating complete diets. Additionally, the copper contribution from treats and other daily supplements needs to be accounted for. Dietary copper sufficiency cannot be evaluated based on calculated copper concentration alone. There are important nutrient interactions that need to be accounted for. Zinc, iron, molybdate, and sulfur are all known to antagonize the amount of bioavailable copper. Zinc is a potent inducer of MT production that will preferentially bind and sequester Cu^+^ and, thus, increase the risk of a zinc-induced copper deficiency, regardless of dietary copper concentration. As mentioned earlier, homeostatic iron maintenance requires copper. However, excess dietary iron can act as an antagonist to copper, thus complicating this nutrient interaction. A study in rats revealed that high dietary iron can cause copper-deficient anemia via disturbances in copper utilization after it has been absorbed (Ha et al., 2017). Additionally, Schultheiss et al. (2002) found that dogs with increased hepatic iron concentrations also had elevated liver copper concentrations. While causation for which mineral may be driving the response cannot be determined, at the very least there appears to be a correlation between iron and copper hepatic accumulation. The basal ingredient iron contribution is elevated in raw meat and grain-free dog diets and is, therefore, an important consideration when determining copper supplementation in these formulations.

The concomitant increase in iron and copper concentrations was observed and compared among dogs with varying degrees of liver lesions (Schultheiss et al., 2002) and underlines the possibility of other mineral contributions to hepatic pathologies. Although there are few data about the interaction of copper and lead, Gori et al. (2021) observed increased liver lead concentrations in dogs with liver copper concentrations above 400 ppm (dry weight basis). Similar to the iron and copper relationship mentioned earlier, there is not sufficient evidence to determine causation versus correlation, but these results emphasize the need for further investigation of the overall nutrient status of dogs suffering from CAH.

Excess molybdate and sulfur can be antagonistic to copper bioavailability. These nutrients can create complexes that bind copper and render it unavailable to the animal. While this may be of greater concern in ruminant animals due to increased production of these complexes in the rumen, it is worthy to note and be aware of when formulating monogastric animal diets, especially when determining sulfur-containing amino acid inclusion.

### Assessing Copper Status

Formulating diets with adequate but not excess copper is made more complex by the lack of reliable, non-invasive copper status biomarkers. Currently, there are no copper status biomarkers for dogs; therefore, liver biopsy is necessary and considered the gold standard for copper toxicosis diagnosis (Fieten et al., 2012). However, there is evidence to support the potential variation in liver copper concentrations based on biopsy type and specimen size (Johnston et al., 2009) and, therefore, caution should be taken when making comparisons among studies. Current recommendation ranges used to determine potential copper toxicity are based on liver copper concentrations expressed on a dry weight basis. Therefore, it is difficult to make comparisons and conclusions from studies based on liver copper concentrations expressed on a wet weight basis (Paβlack et al., 2015; Cedeño et al., 2016).

Cedeño et al. (2020) recently reported that serum copper was increased in dogs with hepatic disease and inflammatory infections. However, serum copper concentrations do not correlate with hepatic copper concentrations in dogs (Dirksen and Fieten, 2017) and, therefore, caution should be taken when using this biomarker to evaluate risk for or diagnosis of liver pathologies.

While alanine aminotransferase and alkaline phosphatase are the most common biomarkers used to indicate hepatocellular injury, they are not usually altered until later stages of hepatopathy when liver damage has begun and may be affected by multiple physiological disturbances. Given these factors, they are not effective at detecting subclinical copper imbalances and, therefore, may not allow detection before irreversible liver damage has occurred (Dirksen and Fieten, 2017).

### Copper Sources

The most common sources of copper used in mineral premixes added to complete diets are copper sulfate or organic sources, such as amino acid-chelated copper. For reference, the term “organic” is used herein and is interchangeable with “chelated” as is most commonly used in companion animal nutrition. Historically, copper oxide was used, but due to its extremely low availability to the animal (Ledoux et al., 1991), it is no longer recommended as a supplemental source of copper. The most common inorganic sources of Cu include sulfates and carbonates. When compared to the bioavailability of reagent grade copper acetate (100%) in a chick bioassay, copper sulfate and carbonate were 88.5% and 54.3% bioavailable, respectively, while copper oxide was determined to be less than 1% bioavailable to the chick (Ledoux et al., 1991). A similar pattern of bioavailability from these inorganic Cu sources was observed in ruminant species (Ledoux et al., 1995).

The increased use of more bioavailable sources of copper supplements in combination with higher basal ingredient contributions of copper has been speculated as being one of the main driving forces for increased CAH cases (Center et al., 2021). However, emphasis should be placed on an effective and reliable supplemental source due to the high potential variability and inconsistency in diet ingredient copper contributions. This will allow nutritionists to include minimal supplemental concentrations to ensure the dog’s requirements are met while also reducing the need to over-formulate copper in diets, thus reducing the risk of toxicity.

The currently available data suggest organic forms of supplemental Cu do not pose an increased risk of toxicity, as measured by liver copper concentrations. As mentioned previously, ruminant species tend to be at a higher risk for copper toxicity and, therefore, serve as a good model to evaluate potential issues among different forms of supplemental Cu. In a study comparing the effect of supplemental copper source and feeding regimen in sheep fed equivalent levels of copper as Cu sulfate or Cu-lysine complex, sheep fed Cu-lysine had significantly less final and overall change from initial liver copper concentrations, regardless of feeding regimen (Luo et al., 1996). Additionally, no differences in liver copper concentrations were detected in dairy cattle fed equivalent concentrations of Cu sulfate compared to a Cu-lysine complex (Chase et al., 2000).

There are data in monogastric species to suggest no increased risk of toxicity due to organic supplemental forms of copper. Guo et al. (2001) evaluated different forms of organic copper (proteinates, lysine complex, and amino acid chelate) in two different breeds of chickens. Overall, they observed slight increases in the bioavailability of copper from the organic sources compared to Cu sulfate (set at 100% bioavailable) as measured by liver copper concentrations, but there were no signs of toxicity reported (Guo et al., 2001). More importantly, the authors noted the different magnitude of response between the two breeds that highlights the inherent limitations of species, breed, and experimental conditions when determining relative bioavailability (Guo et al., 2001). The growth-promoting characteristics of increased copper supplementation were compared in pigs fed two equivalent concentrations of Cu sulfate or Cu-lysine complex. Like the other studies mentioned above, there was no observed increased risk of toxicity in pigs fed the organic Cu-lysine form. Averaged across four different experiments, pigs fed Cu-lysine had lower liver copper concentrations compared to pigs fed Cu sulfate, (Coffey et al., 1994).

### Collaborative Next Steps for Optimizing Canine Health and Longevity

Copper metabolism is a complex physiological process and homeostatic regulation is influenced by multiple factors including, but not limited to, variable copper bioavailability across and within ingredients, stage of development or reproduction, physiological stressors such as inflammation or immune challenges, and sufficient protocols for monitoring copper status. Canine nutrition is further complicated by a wide variety and quality of dietary ingredients and nutritional profiles of commercially available diets.

There is no current research evidence to suggest that the minimum total dietary copper recommendations provided by NRC and AAFCO are insufficient to meet basic physiological needs. However, this is assuming the previously mentioned factors that affect copper metabolism are not perturbed. The uncertainties around dietary ingredient copper contribution and the bioavailability of the copper present often leads to the common practice of formulating diets that all too often exceed those recommended concentrations due to the desire to provide a safety net to avoid a potential deficiency. However, this comes with a risk for nutrients such as copper that are more vulnerable to becoming toxic.

There are multiple opportunities for collaboration among veterinarians, nutritionists, and pet food manufacturers to meet the shared goal of providing pet parents dog food options they can be confident are supplying their dog the necessary nutrients to support an active and healthy life. Next steps to reach those goals might include:

1. Increased emphasis on creating a database that provides average concentrations of copper in common dog diet ingredients that would increase the industry’s knowledge about variable basal ingredient copper contributions in complete diets. Increased testing and transparency of total copper concentrations in dog food would benefit companion animal industry professionals and pet parents.
2. Pet parent education about breeds at higher risk of copper toxicity.
3. Support for copper bioavailability research of individual dog diet ingredients as well as effects of ingredient combinations on bioavailability.
4. Utilization of reliable, consistent, high-quality copper sources in mineral premixes at minimal inclusion concentrations (7 ppm Cu/kg diet).

## CONCLUSIONS

There are many questions that remain regarding copper metabolism in dogs that will most likely continue to evolve as trends in canine nutrition change. Future research efforts should be placed on discovering reliable, non-invasive methods for evaluating copper status that are sensitive enough to detect imbalances prior to liver damage as well as increasing knowledge about variation in ingredient copper bioavailability in dogs. Complex factors such as genetic predisposition and unpredictable physiological stressors need to be considered as well.
